# Influence of Age-Related Complications on Clinical Outcome in Patients With Small Ruptured Cerebral Aneurysms

**DOI:** 10.3389/fneur.2020.00131

**Published:** 2020-03-05

**Authors:** Jianfeng Zheng, Xiaochuan Sun, Xiaodong Zhang

**Affiliations:** Department of Neurosurgery, The First Affiliated Hospital of Chongqing Medical University, Chongqing, China

**Keywords:** small ruptured aneurysm, age, complication, treatment, outcome

## Abstract

**Background:** Small ruptured cerebral aneurysms (≤5 mm) account for the majority of aneurysmal subarachnoid hemorrhages, and its clinical management remains a challenge. The aim of this study was to identify the effect of age-related complications on the outcome of patients with small ruptured aneurysm.

**Methods:** A retrospective review was performed in patients with small ruptured aneurysms who underwent microsurgical clipping or endovascular coiling from September 2012 to December 2018. According to their ages, the patients were divided into the elderly group and the non-elderly group. Baseline characteristics, clinical complications, and outcome of patients were analyzed between the two groups. A multivariate logistic regression analysis was used to determine the risk factors associated with the poor outcome of the elderly patients.

**Results:** In the elderly group, 83 patients were treated with clipping and 50 were treated with coiling. In the non-elderly group, 188 patients were treated with clipping and 117 were treated with coiling. The incidence of neurological complications with neurologic deficit in the elderly group was significantly higher compared with that in the non-elderly group (*P* = 0.006). The elderly patients had a longer hospital stay (*P* = 0.032) and a poorer outcome (*P* = 0.001) compared with the non-elderly patients. A multivariate analysis showed that irregular aneurysms (*P* = 0.045) and ischemic events (*P* < 0.001) were independent risk factors associated with poor outcome in the elderly.

**Conclusions:** Neurological complications with neurologic deficit, especially ischemic complications, were clearly more common in the elderly patients. Irregular small aneurysms or postoperative ischemic events should be paid attention as the higher risk of poor outcome in the elderly.

## Introduction

A growing number of patients with aneurysmal subarachnoid hemorrhage (aSAH) caused by small ruptured cerebral aneurysms have been treated with microsurgical clipping or endovascular coiling (1–3). Compared with the younger patients, the elderly patients may have more underlying comorbidities, poorer physical condition, and higher operation risk (4–6). It is generally agreed that the outcome of the patients is largely determined by the severity of the initial aSAH and subsequent complications associated with neurologic deficit (7, 8). Old age is reported to be one of the significant risk factors that affect the severity of aSAH (7, 9, 10), and the elderly patients with poor cerebrovascular reserve function are more likely to suffer from severe neurological dysfunction after aSAH. Few previous studies have focused on comparing the clinical outcome of patients with small ruptured aneurysms according to age, and most of these previous studies have focused on the treatment outcome of coiling or clipping for patients with small ruptured aneurysms (3, 11–14). It is unclear whether age itself increases the risk of complications in patients during small cerebral aneurysm treatment. Therefore, this study was a desire to investigate the incidence of complications in elderly patients treated for small ruptured aneurysms and to determine whether age-related complications affected the treatment outcome.

## Methods

We retrospectively analyzed the clinical record of 438 patients with small ruptured cerebral aneurysms who were treated with microsurgical clipping or endovascular coiling from September 2012 to December 2018. All of the patients were divided into two groups according to age: the elderly group (those 60 years of age or older) and the non-elderly group (those younger than 60 years). The data of each patient were collected in detail including age, sex, smoking history, drinking history, hypertension, diabetes, cardiopulmonary disease, clinical grade, location, number and shape of aneurysm, treatment modality, clinical complications, and outcomes.

Three-dimensional (3D) rotational angiography was used to examine small aneurysms in all of the patients, and the maximum diameter of the aneurysms as measured by 3D angiography was ≤5 mm. With the use of 3D rotational angiography, the shape and the location of the aneurysms were determined by an experienced neuroradiologist. For patients with multiple small aneurysms, the most likely source of bleeding can be judged by the subarachnoid hemorrhage (SAH) distribution on a CT scan.

Informed consent for the operation was signed after full consideration by the patient or his or her family. The modality of treatment for small aneurysms was determined by the cerebrovascular group composed of experienced neurointerventionists and neurosurgeons. The neurological complications were assessed by two professional neurosurgeons; hemorrhagic event, ischemia event, hydrocephalus, and seizure were recorded. Bleeding events include intraoperative bleeding and postoperative hematoma. Ischemic events include intraoperative thrombosis and postoperative delayed cerebral ischemia. Delayed cerebral ischemia was defined as new or worsening neurological deficits after the exclusion of other possible causes. Hydrocephalus was defined as neurological deterioration accompanied by an enlargement of the temporal horns of more than 2 mm. Cardiopulmonary complications were evaluated by the relevant department physicians, such as arrhythmia, pulmonary infection, and pulmonary embolism. Before discharge, CT angiography or magnetic resonance angiography was performed to evaluate the degree of small aneurysm occlusion and the patency of the parent artery. Each patient's follow-up information was supplemented by imaging reviews and telephone interviews. Based on the Glasgow Outcome Scale (GOS) at the last follow-up, the outcome of each patient was rated and then classified into good outcome (GOS scores of 4 or 5) or poor outcome (GOS scores of 1–3).

### Statistical Analysis

All data in this study were analyzed using SPSS V.22 software (IBM Corp., Armonk, New York, USA). Data related to categorical variables are presented as percentages, and Fisher exact test or Pearson χ^2^ test was used to compare female, smoking, drinking, hypertension, diabetes, cardiopulmonary diseases, clinical grade, location of aneurysms, irregular and multiple aneurysms, complications, and outcome. Data related to continuous variables are presented as mean ± SD, and Student *t*-test or Mann–Whitney *U*-test was used to compare age and hospital days. All clinical variables with *P* < 0.1 were entered into the multivariate analysis models, and odds ratios (ORs) with 95% confidence intervals (CIs) were calculated.

## Results

A total of 133 (30.4%) patients were in the elderly group and 305 (69.6%) were in the non-elderly group, with an average age of 53 years (range, 23–77 years). In the elderly group, the prevalence of hypertension (60.9 vs. 42.3%; *P* < 0.001), diabetes (11.3 vs. 3.9%; *P* < 0.001), and cardiopulmonary disease (12.0 vs. 2.6%; *P* < 0.001) were significantly increased. More Hunt–Hess grade 4 or 5 cases were observed in the elderly group (21.8 vs. 9.8%; *P* = 0.001), but there was no significant difference in the modified Fisher grade 3 or 4. The other baseline characteristics of the two groups are shown in Table 1.

**Table 1:** Baseline characteristics of patients with small ruptured aneurysms.

	**Overall**	**Non-elderly (*n* = 305)**	**Elderly (*n* = 133)**	***P*-value**
Age (year)	53.4 ± 9.8	48.2 ± 6.5	65.3 ± 4.1	<0.001
Sex (female)	310 (70.8%)	210 (68.9%)	100 (75.2%)	0.209
Tobacco smoking	94 (21.5%)	69 (22.6%)	25 (18.8%)	0.448
Alcohol consumption	76 (17.4%)	58 (19.0%)	18 (13.5%)	0.173
Hypertension	210 (47.9%)	129 (42.3%)	81 (60.9%)	<0.001
Diabetes	27 (6.2%)	12 (3.9%)	15 (11.3%)	<0.001
Cardiopulmonary diseases	24 (5.5%)	8 (2.6%)	16 (12.0%)	<0.001
Hunt–Hess Grade 4 or 5	59 (13.5%)	30 (9.8%)	29 (21.8%)	0.001
Modified Fisher Grade 3 or 4	180 (41.1%)	120 (39.3%)	60 (45.1%)	0.291

*Results with P < 0.05 were considered to indicate statistical significance*.

There were 158 small ruptured aneurysms located in the anterior communicating artery (AcomA), 145 in the posterior communicating artery (PcomA), 55 in the middle cerebral artery (MCA), 17 in the anterior cerebral artery (ACA), 52 in the internal carotid artery (ICA), and 11 in the posterior circulation (PC). The non-elderly patients have more ruptured small aneurysms in the location of the AcomA (39.3 vs. 28.6%; *P* = 0.031), while the elderly patients have more ruptured small aneurysms in the location of the PcomA (43.6 vs. 28.5%; *P* = 0.003). It was observed that the elderly patients had significantly more multiple aneurysms than the non-elderly patients (22.6 vs. 11.5%; *P* = 0.005). The irregularly shaped aneurysms were similar between the two groups (Table 2).

**Table 2:** Location and characteristics of the small ruptured aneurysms.

	**Overall**	**Non-elderly (*n* = 305)**	**Elderly (*n* = 133)**	***P*-value**
**Aneurysm location**				
AcomA	158 (36.1%)	120 (39.3%)	38 (28.6%)	0.031
PcomA	145 (33.1%)	87 (28.5%)	58 (43.6%)	0.003
MCA	55 (12.6%)	35 (11.5%)	20 (15.0%)	0.347
ACA	17 (3.9%)	15 (4.9%)	2 (1.5%)	0.109
ICA	52 (11.9%)	39 (12.8%)	13 (9.8%)	0.424
PC	11 (2.5%)	9 (3.0%)	2 (1.5%)	0.516
**Aneurysm characteristics**				
Irregular	67 (15.3%)	46 (15.1%)	21 (15.8%)	0.885
Multiple	65 (14.8%)	35 (11.5%)	30 (22.6%)	0.005

*AcomA, anterior communicating artery; PcomA, posterior communicating artery; MCA, middle cerebral artery; ACA, anterior cerebral artery; ICA, internal carotid artery; PC, posterior circulation. Results with P < 0.05 were considered to indicate statistical significance*.

### Clinical Complications and Outcome

There was no significant difference in treatment modality and intraoperative strategy between the elderly group and the non-elderly group (Table 3). Common neurological complications included hemorrhage events in 13 cases (3.0%), ischemic events in 98 cases (22.4%), hydrocephalus in 69 cases (15.8%), and seizure in 15 cases (3.4%). Hemorrhage events included intraoperative rebleeding in six patients (1.4%) and postoperative rebleeding in seven patients (1.6%). The ischemic events included intraoperative thrombosis in three patients (0.7%) and postoperative delayed cerebral ischemia in 95 (21.7%) patients. Temporary clipping was performed in 80 cases (31.1%) and was not associated with hemorrhage events (*P* = 0.238) or ischemic events (*P* = 0.963). Stent-assisted coiling was performed in 113 (25.8%) patients and balloon-assisted coiling was performed in four patients (1.0%). Neither stent-assisted nor balloon-assisted strategy was associated with hemorrhage events (*P* = 0.389 and *P* = 1.000, respectively) or ischemic events (*P* = 0.106 and *P* = 0.999, respectively). Common cardiopulmonary complications included arrhythmia in 30 cases (6.8%), pulmonary infection in 147 cases (33.6%), and pulmonary embolism in five cases (1.1%).

**Table 3:** Complications and outcomes of patients with small ruptured aneurysms.

	**Overall**	**Non-elderly (*n* = 305)**	**Elderly (*n* = 133)**	***P*-value**
Clipping	271 (61.9%)	188 (61.6%)	83 (62.4%)	0.915
Coiling	167 (38.1%)	117 (38.4%)	50 (37.6%)	
Balloon-assisted	4 (1.0%)	2 (0.6%)	2 (1.6%)	0.516
Stent-assisted	113 (25.8%)	78 (25.6%)	35 (26.3%)	0.906
Temporary clipping used	80 (18.3%)	58 (19.0%)	22 (16.5%)	0.592
Neurological complications	113 (25.8%)	71 (23.3%)	42 (31.6%)	0.075
Hemorrhagic event	13 (3.0%)	10 (3.3%)	3 (2.3%)	0.762
Ischemic event	98 (22.4%)	60 (19.7%)	38 (28.6%)	0.046
Hydrocephalus	69 (15.8%)	44 (14.4%)	25 (18.8%)	0.256
Seizure	15 (3.4%)	10 (3.3%)	5 (3.8%)	0.780
Cardiopulmonary complications	166 (36.5%)	101 (33.1%)	59 (44.4%)	0.031
Arrhythmia	30 (6.8%)	16 (5.2%)	14 (10.5%)	0.062
Pulmonary infection	147 (33.6%)	90 (29.5%)	57 (42.9%)	0.008
Pulmonary embolism	5 (1.1%)	1 (0.3%)	4 (3.0%)	0.031
Length of stay (day)	21.1 ± 10.9	20.3 ± 9.0	23.2 ± 14.1	0.032
Good outcome (GOS 4 or 5)	386 (88.1%)	280 (91.8%)	106 (79.7%)	0.001
Poor outcome (GOS 1 or 3)^a^	52 (11.9%)	25 (8.2%)	27 (20.3%)	

*GOS, Glasgow Outcome Scale. Results with P < 0.05 were considered to indicate statistical significance*.

a *After adjustment for smoking, hypertension, cardiopulmonary disease, Hunt–Hess grade, modified Fisher grade, multiple aneurysms, irregular aneurysms, and treatment modality, a multivariate analysis showed that the elderly was independently associated with poor outcome [OR, 2.025 (1.015–4.040); P = 0.045]*.

**Figure 1:**
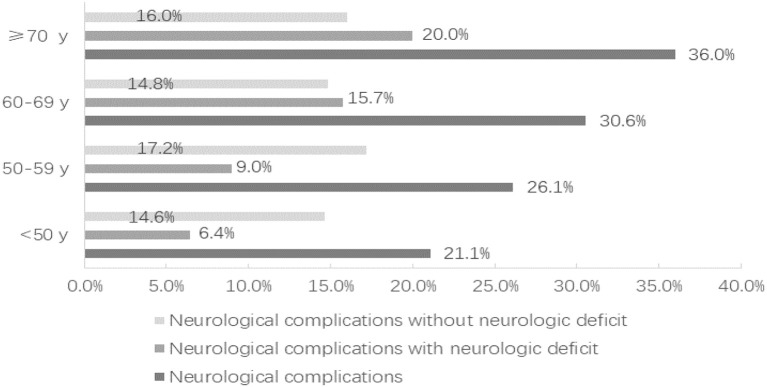
The distribution of neurological complications according to the age of the patients, divided into those with neurologic deficit and those without neurologic deficit.

Compared with the non-elderly group, the incidence of ischemic events (28.6 vs. 19.7%; *P* = 0.046), cardiopulmonary complications (44.4 vs. 33.1%; *P* = 0.031), and pulmonary infections (42.9 vs. 29.5%; *P* = 0.008) was significantly higher in the elderly group. The total neurological complications (31.6 vs. 23.3%; *P* = 0.075) and the neurological complications without neurological deficits (15.0 vs. 15.7%; *P* = 0.887) did not vary significantly with the increase of patient age (Figure 1). However, compared with the non-elderly patients, the more elderly patients suffered from neurological complications with neurologic deficit (16.5 vs. 7.5%; *P* = 0.006). As a result of complications, the length of stay in the elderly group was significantly longer than that in the non-elderly group (23.2 ± 14.1 vs. 20.3 ± 9.0; *P* = 0.032) (Table 3).

**Figure 2:**
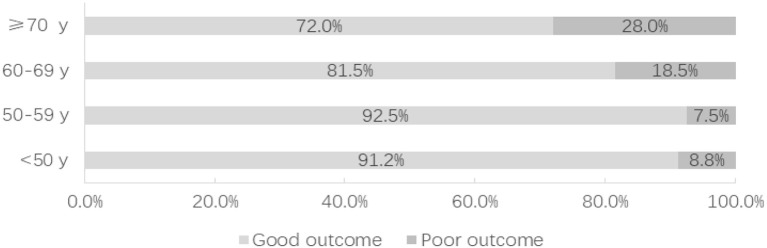
The distribution of functional outcome according to the age of the patients, divided into those with good outcome and poor outcome.

After 3–60 months of follow-up (17 months on average), 88.1% of the patients had a good outcome (GOS 4 or 5). Compared with the non-elderly group, poor outcome occurred at a significantly greater rate in the elderly group (20.3 vs. 8.2%; *P* = 0.001). Whether treated by microsurgical clipping or endovascular coiling, the outcome of the patients with small ruptured aneurysms was worse with the increase of age (Figure 2). Only the elderly patients were considered, and the risk factors of poor outcome were analyzed. A univariate analysis showed that hypertension (*P* = 0.022), Hunt–Hess grade 4 or 5 (*P* < 0.001), modified Fisher grade 3 or 4 (*P* = 0.001), irregular aneurysms (*P* < 0.001), multiple aneurysms (*P* = 0.019), ischemic events (*P* < 0.001), seizure (*P* = 0.047), and cardiopulmonary complications (*P* = 0.033) were significantly associated with the poor outcome of the elderly patients with small ruptured aneurysm. A multivariate analysis showed that irregular aneurysms [OR, 4.465 (1.032–19.310); *P* = 0.045] and ischemic events [OR, 13.532 (4.128–44.364); *P* < 0.001] were independent predictive factors of poor outcome in the elderly patients (Table 4).

**Table 4:** Risk factors of poor outcome in elderly patients with small ruptured aneurysms.

	**Univariate analysis**		**Multivariable analysis**	
	***P*-value**	**OR (95% CI)**	***P*-value**	**OR (95% CI)**
Hypertension	0.022	3.373 (1.187–9.584)	0.404	–
Hunt–Hess Grade 4 or 5	<0.001	8.214 (3.194–21.126)	0.248	–
Modified Fisher Grade 3 or 4	0.001	4.714 (1.830–12.142)	0.487	–
Irregular aneurysms	<0.001	6.600 (2.412–18.062)	0.045	4.465 (1.032–19.310)
Multiple aneurysms	0.019	2.956 (1.191–7.336)	0.535	–
Ischemic event	<0.001	18.324 (6.443–52.108)	<0.001	13.532 (4.128–44.364)
Seizure	0.047	6.500 (1.029–41.068)	0.848	–
Cardiopulmonary complications	0.033	2.590 (1.082–6.200)	0.637	

*Variables with P < 0.1 according to univariate analysis were entered into the multivariate analysis model. OR, odds ratio; CI, confidence interval. Results with P < 0.05 were considered to indicate statistical significance*.

## Discussion

We presented a large number of patients with small ruptured cerebral aneurysms to compare the effect of age-related complications on the functional outcome. The incidence of neurological complications associated with neurological deficit, especially ischemic events, was obviously higher in the elderly patients than in the non-elderly patients. As a result of age-related complications, the outcome of the elderly patients is significantly worse compared with that of the non-elderly patients.

With the advances in medical imaging facilities and techniques, a growing number of small cerebral aneurysms in the world have been detected and treated (1–3, 7, 11, 14). A retrospective study of 223 small unruptured aneurysms by Oishi et al. (11) reported that endovascular treatment is effective with low morbidity and mortality. Feng et al. (14) thought that the coil embolization of small unruptured aneurysms can be performed safely and achieve a low rate of recurrence. Chalouhi et al. (15) reported that patients with small ruptured aneurysms were treated with a pipeline embolization device, and these patients had a good outcome. Although endovascular coiling was technically feasible, smaller aneurysms were reported to be significantly associated with a higher risk of procedural aneurysm rupture (16). Mitchell et al. (17) believed that a small aneurysm size is a significant risk factor for intraoperative rupture in ruptured aneurysms rather than in unruptured aneurysms. In our study of 438 small ruptured cerebral aneurysms, six patients suffer from intraoperative rebleeding. The incidence of intraoperative aneurysm rupture was similar between the elderly and the non-elderly patients, and these did not lead to serious neurological dysfunctions.

However, due to the smaller size and the weaker wall of the aneurysm, and the more difficult operation, the risk of postoperative ischemic complications was significantly increased. Delayed cerebral ischemia is reported to occur in about 30% of patients with aSAH and leads to higher morbidity and mortality (18, 19). Our study on small ruptured aneurysm showed that the incidence of delayed cerebral ischemia was 21.7%, which was obviously higher in the elderly patients than that in the non-elderly patients. Wachter et al. (20) believes that the elderly patients seem to be more vulnerable to aSAH, plus the complications are more problematic. The severe aSAH after rupture together with the subsequent operation on the surrounding anatomical structures such as parent arteries, perforators, brain parenchyma, and veins will cause cerebral vasospasm, which can be further aggravated by the decline of the cerebrovascular regulatory function in the elderly. In our study, although there was no significant difference in the high modified Fisher grade, the high Hunt-Hess grade in the elderly patients was more significant than in the non-elderly patients. This may mean that the elderly patients with poor cerebrovascular reserve capacity are more likely to suffer from severe brain damage after aSAH, even if the aneurysm size is small. The elderly patients have more comorbidities such as hypertension, diabetes, and cardiopulmonary disease, which makes the treatment risk of this special elderly population higher. Differently from our results, another aSAH study (20) reported a lower incidence of delayed cerebral ischemia in the elderly patients than in the non-elderly patients due to arteriosclerosis. The outcome of the study by Lai et al. (21) is consistent with our results that older women are at obviously greater risk of cerebral vasospasm, cerebral infarction, and clinical deterioration. Since the majority (75.2%) of the elderly patients in our study are female, female hormones could play a very important role in the development of ischemic complications in aSAH patients (21).

The total mortality rate of our patients with small ruptured aneurysms was 3.2%, higher than 0.91% of patients with small unruptured aneurysms as reported by Feng et al. (14). It should be noted that a more aggressive treatment performed before the rupture status of small cerebral aneurysms may reduce the mortality, but older age also needs to be considered, which is a very important factor affecting the incidence of complications associated with functional outcome. A study of in-patient samples (22) showed that the mortality rate of patients over 65 years old was obviously higher than that of patients younger than 65 years. Several other studies (23–25) have suggested that the outcome was worse with the increase of age in patients treated with clipping or coiling, but no emphasis has been placed on the influence of age-related complications on functional outcome. Similarly, in our study, the poor outcome occurred at an obviously greater rate in the patients aged 60 years and over compared with the patients under 60 years old, while the outcome of patients over 70 years old was the worst. The neurological complications have a greater impact on the neurological function of the elderly patients.

In the past 10 years of cerebral aneurysm research, the proportion of small ruptured aneurysms has increased to nearly 50% (26–28). Kim et al. believed that a sidewall aneurysm found with multiple aneurysms or an AcomA aneurysm found from a patient with hypertension may be prone to rupture at smaller sizes. In a prospective study in Japan (29), patient age and PcomA were reported to be significant risk factors for rupture in the elderly patients. Similarly, our results show that small ruptured cerebral aneurysms at the location of the PcomA are more common and the proportion of multiple aneurysms is higher in the elderly patients compared with those in the non-elderly patients. A recent survey (30) indicated that 42–76% of surgeons suggested treating the small anterior circulation aneurysms with a SAH history and most of the surgeons stop treating the unruptured aneurysms in patients who are 70–80 years old. Therefore, in order to avoid over-intervention as well as in consideration of the higher aneurysm rupture risk and the age-related complication risk, more preventive treatment is suitable for the younger patients with small unruptured communicating segment (PcomA/AcomA) aneurysms or with multiple aneurysms.

## Limitations

There are several limitations in the current study. The interpretation of these final conclusions needs to take into account our single-center retrospective design. Firstly, since there is no prospective randomized intervention for the small ruptured aneurysms, potential selection bias may affect the efficacy of the treatment. However, the aim of our study was to ascertain the relationship between age-related complications and clinical outcomes rather than simply comparing the outcomes of the different treatment modalities. Secondly, due to the underdevelopment of the economy and the medical level in Western China, fewer patients over the age of 70 received operation and none over the age of 80 received intervention. Thirdly, we define the elderly as patients aged 60 and over, while other studies have used a different age cutoff, so the overall morbidity and mortality may be different from other research data.

## Conclusions

This study provides some useful data for the clinical management of elderly patients with small ruptured aneurysm. Based on our findings, we conclude that neurological complications associated with neurologic deficit were clearly more common in the elderly patients than in the non-elderly patients. The preoperative irregular small aneurysms or the postoperative ischemic events should be paid attention as the higher risk of poor outcome in elderly patients.

## Data Availability Statement

The raw data supporting the conclusions of this article will be made available by the authors, without undue reservation, to any qualified researcher.

## Ethics Statement

The studies involving human participants were reviewed and approved by Ethics committee of the First Affiliated Hospital of Chongqing Medical University. The patients/participants provided their written informed consent to participate in this study.

## Author Contributions

JZ was involved in the acquisition of the data, the analysis and interpretation of the data, the drafting of the article, and the final approval of the version to be published. XS and XZ critically revised the article for intellectual content and gave final approval of the version to be published.

### Conflict of Interest

The authors declare that the research was conducted in the absence of any commercial or financial relationships that could be construed as a potential conflict of interest.
